# Transnasal endoscopic removal of a pituicytoma: a case report

**DOI:** 10.3389/fonc.2025.1550272

**Published:** 2025-11-03

**Authors:** Daniyar Teltayev, Torebek Tursynbekov, Khalit Mustafin, Almas Kydyrov, Tatyana Litvinova, Nurali Ashirov, Ainur Turzhanova, Makar Solodovnikov, Serik Akshulakov

**Affiliations:** ^1^ Department of Minimally Invasive Neurosurgery, National Centre for Neurosurgery, Astana, Kazakhstan; ^2^ Department of Research Management, National Centre for Neurosurgery, Astana, Kazakhstan; ^3^ National Centre for Neurosurgery, Astana, Kazakhstan

**Keywords:** pituicytoma, transsphenoidal surgery, sellar-suprasellar tumor, endoscopic neurosurgery, histology 1

## Abstract

Pituicytoma is an exceedingly rare tumor of the pituitary gland, classified as WHO grade 1. Its presentation varies widely, often mimicking other pituitary or sellar region tumors. This report discusses a unique case of pituicytoma in a young female presenting with a combination of visual disturbances, infertility, and endocrine dysfunction, emphasizing the role of multidisciplinary management and minimally invasive surgical techniques.

## 1 Introduction

Pituicytomas are extremely rare tumors of the chiasmal-sellar region, originating from the cells of the pituitary stalk or the posterior lobe of the pituitary gland (1). These tumors were first described in the literature in 1955. The World Health Organization (WHO) classified pituicytomas in 2021 as grade I glial neoplasms, comprising three distinct histological subtypes: pituicytoma, granular cell tumor, and spindle cell oncocytoma (2–6).

Pituicytoma is poorly understood neoplasms of the sellar region. It was only in the 2007 WHO Classification of Tumors of the Central Nervous System that pituicytoma was recognized as a distinct tumor, separate from granular cell tumors. The 2007 edition also introduced spindle cell oncocytoma as a new entity in the differential diagnosis of sellar region neoplasms (2).

Differentiating pituicytomas from other pituitary tumors is challenging due to their lack of specific radiological features (2, 7–9). As a result, tumors in the chiasmal-sellar region are often misdiagnosed as pituitary adenomas. Diagnosis is confirmed based on histopathological findings and immunohistochemical (IHC) studies. Although histologically benign, these tumors are hypervascularized, which complicates surgical removal (1, 10).

Treatment typically involves surgical resection, often resulting in a favorable prognosis. Below, we present a case of pituicytoma with endosupra-laterosellar growth at a stage of moderate clinical decompensation.

## 2 Case report

**Figure 1:**
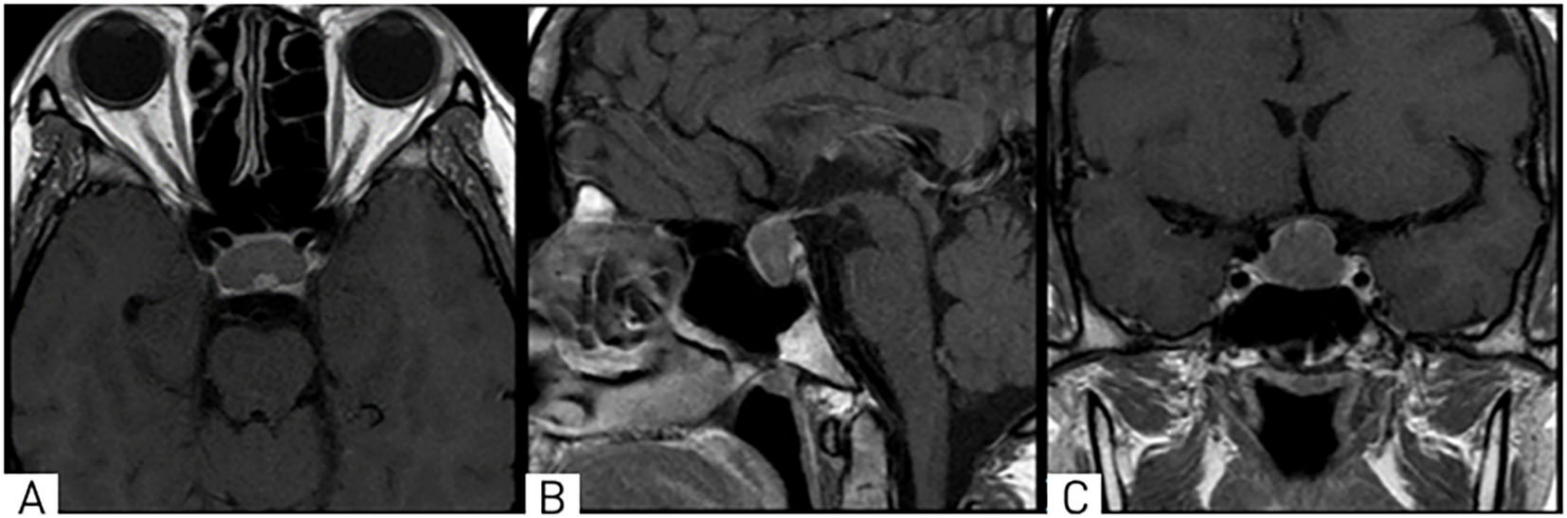
On MRI, T1-weighted images in axial **(A)**, sagittal **(B)**, and coronal **(C)** projections show a well-defined mass in the chiasmal-sellar region with predominantly endosupra-laterosellar growth, compressing the optic chiasm.

A 28-year-old female patient presented to the National Center for Neurosurgery with complaints of headaches, decreased visual acuity, infertility for 4 years, and menstrual irregularities (amenorrhea). An ophthalmologist consultation revealed the following diagnoses: OU - papilledema, OU - peripheral and central chorioretinal degeneration, OU - mild myopia, OU - retinal vascular angiopathy, and OU - ocular hypertension. Brain MRI (Magnetic Resonance Imaging) with contrast was recommended. MRI revealed a mass in the chiasmal-sellar region with predominantly endosupra-sellar and minor lateral-sellar growth (**Figure 1**).

**Figure 2:**
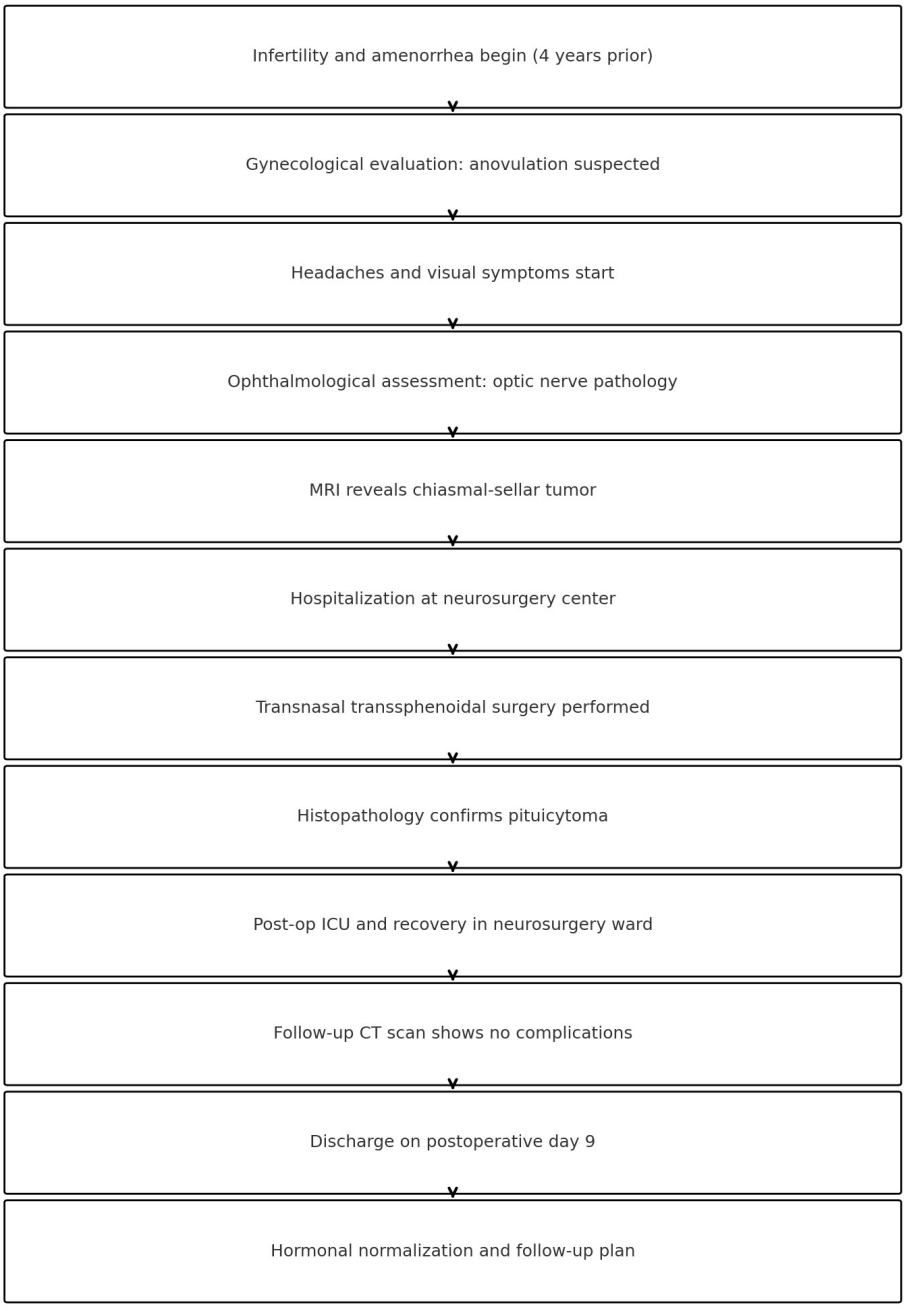
Timeline of key clinical events and interventions throughout the patient’s course.

Due to the complexity and length of the patient’s clinical course, a timeline summarizing the key events is provided (**Figure 2**) to clarify the sequence of diagnostic and therapeutic milestones.

Laboratory tests show signs of hypopituitarism, with decreased levels of prolactin and growth hormone. Immunochemiluminescent hormone analysis: Prolactin: 8.54 ng/ml (reference values: 4.8–23.3 ng/ml), Cortisol: 448.20 nmol/L (reference values: 138–690 nmol/L), TSH: 3.96 mIU/L (reference values: 0.4–4.0 mIU/L), Somatotropin: 0.0595 ng/ml (reference values: <5 ng/ml), IGF-1: 82.90 ng/ml (reference values: 116–358 ng/ml).

Given the tumor’s location, the patient’s age, and the clinical picture, it was decided to perform surgery via a minimally invasive approach – transnasal transsphenoidal resection of the chiasmal-sellar region tumor using the KARL STORZ endoscopic system with 0° and 45° rigid endoscopes. To prevent infectious complications, standard perioperative antibiotic prophylaxis was administered preoperatively. This included intravenous administration of cefuroxime 1500 mg and metronidazole 500 mg, along with the use of a local antiseptic.

Intraoperatively: A gray tumor was found, soft, viscous, jelly-like (partially suctioned, with difficulty), moderately bleeding. The tumor measured approximately 3.0 x 3.0 x 2.0 cm. No obvious signs of cerebrospinal fluid leakage were found. The tumor was completely removed. Part of the tumor was sent for histological examination.

Endoscopic inspection of the chiasmal-sellar region revealed a grayish-yellow, soft, gelatinous tumor with areas of moderate vascularization. The lesion was partially aspirated; however, due to its viscous consistency and vascularity, careful dissection from surrounding structures including the pituitary stalk and optic chiasm was required. Visual control of the tumor margins was achieved. Hemostasis was accomplished using monopolar coagulation in combination with application of a hemostatic agent (Surgicel). No signs of cerebrospinal fluid (CSF) leakage were observed. Skull base reconstruction was performed using a periosteal flap and fibrin glue.

Tumor tissue fragments were fixed in a 10% buffered formalin solution for 24 hours. After traditional processing, histological sections were stained with hematoxylin and eosin. Histological examination was conducted using an OLYMPUS microscope at magnifications of 100x and 200x.

Histological examination of the surgical biopsy material showed that the histological preparations, stained with hematoxylin and eosin, contained fragments of tumor tissue consisting of fairly dense growths of round-oval, elongated, and bipolar spindle-shaped cells amidst homogeneous eosinophilic masses. The nuclei of the cells were hyperchromatic, with mitotic activity. Numerous blood vessels and vascular cavities were visible. Fragments of pituitary adenoma tissue were also observed.

Immunohistochemical analysis was performed according to the standard procedure using the BenchMark XT immunostainer with RTU antibodies: Vim: diffuse positive reaction, S100: diffuse positive reaction, EMA: positive reaction in isolated cells, Ki67: 0-1%, GFAP: focal positive reaction, PRg: negative reaction. The panel of immunohistochemical markers was selected by the histology technician based on the established diagnostic protocol at our institution.

**Figure 3:**
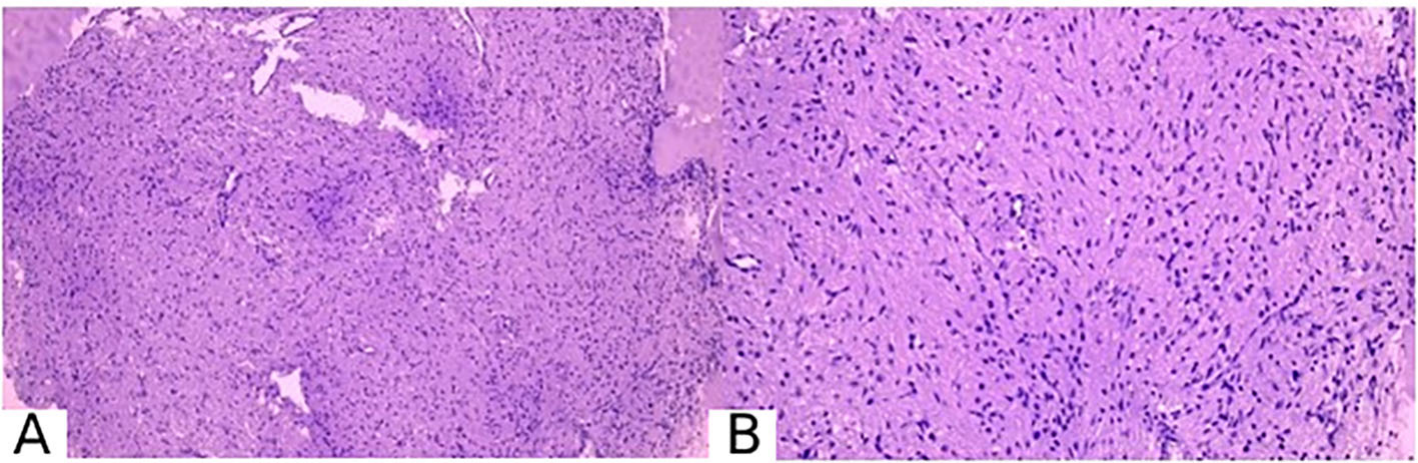
Pathomorphological picture of pituicytoma. **(A)** magnification ×100, **(B)** magnification ×200. Stained with hematoxylin and eosin.

Based on the pathohistological and immunohistochemical findings, the conclusion was as follows: The pathomorphological picture and immunophenotype correspond to small fragments of pituicytoma, WHO grade 1, ICD-O code 9432/1. This is consistent with the clinical and imaging data (**Figure 3**).

**Figure 4:**
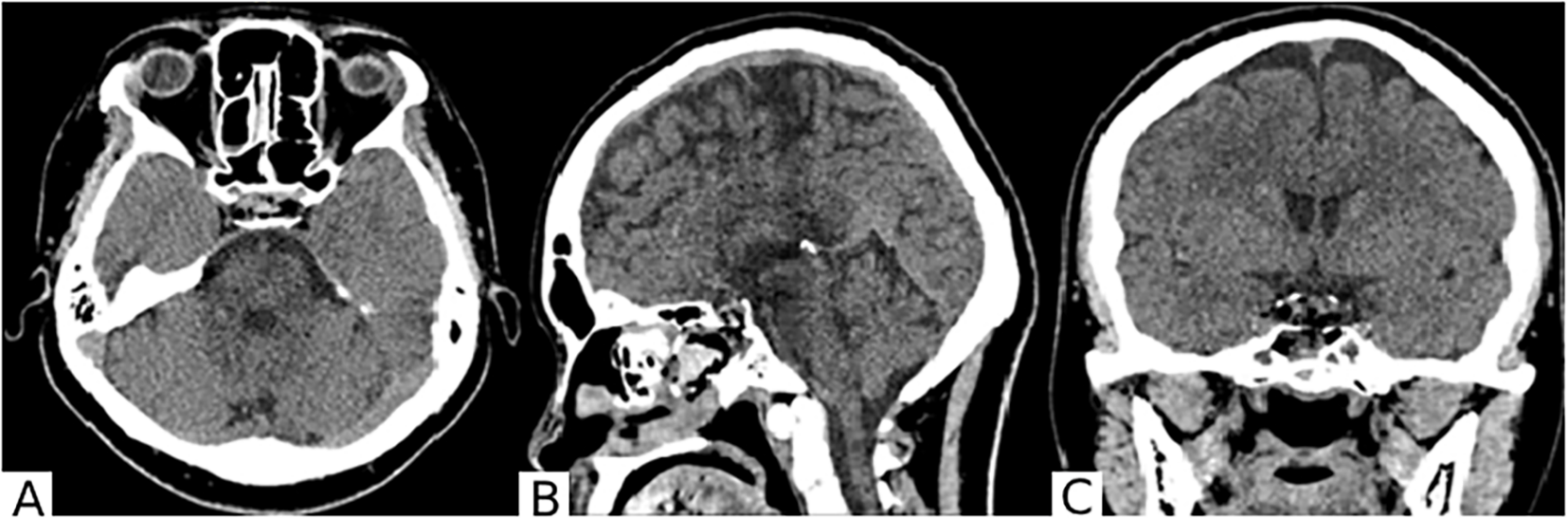
On the brain CT scan in axial **(A)**, sagittal **(B)**, and frontal **(C)** projections after surgery, the surgical site in the chiasmal-sellar region is visible. A small hemorrhagic component is present in the main sinus. The lateral III-IV ventricles are not enlarged. Extracerebral cerebrospinal fluid spaces are unremarkable.

The surgery was performed without complications. The patient was observed for the first 20 hours in the intensive care unit of anesthesiology and resuscitation, followed by transfer to the specialized neurosurgical department. No complications were observed in the postoperative period. A follow-up CT scan of the brain was performed on the first postoperative day (**Figure 4**).

In the postoperative period, antibacterial therapy was continued due to the transnasal surgical approach through the nasal cavity and sphenoid sinus, which are considered potentially contaminated areas and cannot be fully sterilized with antiseptics as is possible with skin. The extended antibiotic regimen was maintained for five days, in accordance with the institution’s internal protocol, to reduce the risk of postoperative infection in surgeries involving mucosal entry. The patient was discharged on postoperative day 9 without clinical complications or complaints.

A repeat hormonal panel was performed, showing a tendency toward normalization of prolactin, TSH, growth hormone, and cortisol levels. No clinical signs of hypopituitarism were observed. Continued dynamic follow-up with hormonal monitoring is planned. The patient is under observation, and an MRI of the brain (at least 1.5 Tesla) with contrast enhancement is planned for 3 and 6 months, followed by a consultation with a neurosurgeon.

## 3 Discussion

This case underscores the importance of recognizing atypical presentations of pituitary tumors. Despite typical clinical manifestations such as headaches, vision impairment, and hormonal dysfunction, these tumors are often mistakenly diagnosed as pituitary adenomas, complicating the choice of treatment strategy.

Pituicytoma is rare neoplasms of the neurohypophysis, characterized by benign behavior and diagnostic challenges. These tumors are often associated with hyperactivity of the adjacent pituitary tissue, leading to clinically uneven hormone secretion. The diagnosis of pituicytoma is challenging when patients exhibit hormonal dysfunction. Several reports have documented cases of pituicytoma with endocrine abnormalities, emphasizing the variable hormonal profiles and the importance of thorough endocrinological workup (8, 11).

In this clinical case, the diagnosis of “Pituicytoma” was established based on histological and immunohistochemical findings. The pathological picture of the tumor, including dense growths of spindle-shaped cells and high vascular density, was confirmed by positive reactions for Vim and S100, which align with current knowledge of gliomas in the neurohypophysis. Immunohistochemical evaluation, beyond confirming the diagnosis, was crucial in excluding other tumors in the chiasmal-sellar region.

An additional histological finding in our case was the presence of fragments resembling pituitary adenoma tissue adjacent to the pituicytoma. While rare cases of concurrent pituitary adenoma and pituicytoma so-called “collision tumors” have been described in the literature (5, 11), we believe that in this case, the observed fragments more likely represent residual adenomatous tissue or a diagnostic artifact rather than a distinct co-existing neoplasm. This interpretation is supported by the absence of clearly separate histological zones and the overlapping immunohistochemical profile (Vim+, S100+, Ki-67 <1%, EMA+ in isolated cells), which argues against two biologically distinct tumors. This highlights the importance of careful pathological examination and multidisciplinary interpretation in sellar region tumors, where tissue overlap may lead to diagnostic challenges.

Surgical treatment was performed using a transnasal transsphenoidal approach. This method demonstrated high efficacy, achieving complete tumor resection with minimal surgical risk, as evidenced by studies reporting high success rates and minimal postoperative complications in similar cases (12–14). The postoperative period was uneventful, highlighting the advantages of the minimally invasive approach for this tumor location.

Special attention was given to the postoperative management. The use of antibiotic therapy was justified considering the potential contamination during surgery. Regular follow-up with MRI of the brain with contrast enhancement is planned to timely detect any recurrence or residual tumor processes.

Despite the benign nature of pituicytoma and successful surgical treatment, long-term radiological follow-up is essential, particularly in cases where the completeness of resection is uncertain. Nevertheless, when gross total resection is achieved, adjuvant therapy is generally not required, as supported by the literature (2, 5). This case emphasizes the importance of a multidisciplinary approach and the standardization of diagnostic and therapeutic protocols for rare neoplasms of the neurohypophysis.

## 4 Limitation

The primary limitation of this report is its focus on a single case. Additionally, as pituicytoma is exceedingly rare, comprehensive conclusions about its management and outcomes are constrained by the lack of extensive comparative data.

## 5 Conclusion

Pituicytoma is rare, non-invasive glial tumors of the chiasmal-sellar region, arising from neurohypophyseal pituitary cells. Surgical resection via a transnasal transsphenoidal endoscopic approach offers a safe and effective treatment. Accurate diagnosis depends on immunohistochemical analysis, which is essential for distinguishing pituicytomas from other sellar region tumors and guiding postoperative management. While long-term follow-up is important, adjuvant therapy is typically unnecessary after complete resection.

## Data Availability

The datasets presented in this article are not readily available because of ethical and privacy restrictions. Requests to access the datasets should be directed to the corresponding author/s.
